# Periodontal Tissues, Maxillary Jaw Bone, and Tooth Regeneration Approaches: From Animal Models Analyses to Clinical Applications

**DOI:** 10.3390/nano8050337

**Published:** 2018-05-16

**Authors:** Fareeha Batool, Marion Strub, Catherine Petit, Isaac Maximiliano Bugueno, Fabien Bornert, François Clauss, Olivier Huck, Sabine Kuchler-Bopp, Nadia Benkirane-Jessel

**Affiliations:** 1 INSERM (French National Institute of Health and Medical Research), UMR 1260, Regenerative NanoMedicine (RNM), FMTS, 67000 Strasbourg, France; s_fareeha_b@hotmail.com (F.B.); marion.strub@chru-strasbourg.fr (M.S.); catherin.petit@gmail.com (C.P.); isaacmaxi@gmail.com (I.M.B.); bornertfabien@gmail.com or fabien.bornert@chru-strasbourg.fr (F.B.); francois_clauss@hotmail.com or francois.clauss@chru-strasbourg.fr (F.C.); huck.olivier@gmail.com or o.huck@unistra.fr (O.H.); nadia.jessel@inserm.fr (N.B.-J.); 2 Faculty of Dentistry, University of Strasbourg (UDS), 8 rue Ste Elisabeth, 67000 Strasbourg, France; 3 Departement of Pediatric Dentistry, Pôle de Médecine et Chirurgie Bucco-Dentaires, Hôpitaux Universitaires de Strasbourg (HUS), 1 place de l’Hôpital, 67000 Strasbourg, France; 4 Department of Periodontology, Pôle de Médecine et Chirurgie Bucco-Dentaires, Hôpitaux Universitaires de Strasbourg (HUS), 1 place de l’Hôpital, 67000 Strasbourg, France; 5 Department of Oral Surgery, Pôle de Médecine et Chirurgie Bucco-Dentaires, Hôpitaux Universitaires de Strasbourg (HUS), 1 place de l’Hôpital, 67000 Strasbourg, France

**Keywords:** bioengineered tooth, BMP-2, cyclosporine A, electrospun polycaprolactone, ibuprofen, innervation, nanoreservoirs, periodontitis

## Abstract

This review encompasses different pre-clinical bioengineering approaches for periodontal tissues, maxillary jaw bone, and the entire tooth. Moreover, it sheds light on their potential clinical therapeutic applications in the field of regenerative medicine. Herein, the electrospinning method for the synthesis of polycaprolactone (PCL) membranes, that are capable of mimicking the extracellular matrix (ECM), has been described. Furthermore, their functionalization with cyclosporine A (CsA), bone morphogenetic protein-2 (BMP-2), or anti-inflammatory drugs’ nanoreservoirs has been demonstrated to induce a localized and targeted action of these molecules after implantation in the maxillary jaw bone. Firstly, periodontal wound healing has been studied in an induced periodontal lesion in mice using an ibuprofen-functionalized PCL membrane. Thereafter, the kinetics of maxillary bone regeneration in a pre-clinical mouse model of surgical bone lesion treated with BMP-2 or BMP-2/Ibuprofen functionalized PCL membranes have been analyzed by histology, immunology, and micro-computed tomography (micro-CT). Furthermore, the achievement of innervation in bioengineered teeth has also been demonstrated after the co-implantation of cultured dental cell reassociations with a trigeminal ganglia (TG) and the cyclosporine A (CsA)-loaded poly(lactic-co-glycolic acid) (PLGA) scaffold in the jaw bone. The prospective clinical applications of these different tissue engineering approaches could be instrumental in the treatment of various periodontal diseases, congenital dental or cranio-facial bone anomalies, and post-surgical complications.

## 1. Introduction

Tooth loss undermines oral health, affecting both function and aesthetics, compromising oral health related quality of life [1]. Periodontitis, a group of inflammatory diseases of infectious origin, is considered as the main cause of tooth loss. It is characterized by progressive destruction of the tooth-supporting tissues (gingiva, cementum, alveolar bone, and periodontal ligament) resulting in gingival bleeding, increased periodontal pocket depth, abscess formation, tooth mobility, and—consequently—tooth loss [2]. Besides the conventionally employed therapy, mainly comprising scaling and root planing to reduce bacterial load, regeneration of destructed tissues is the ultimate objective of periodontal treatment as it has been demonstrated to improve function and long-term retention of the tooth [3]. Currently, for the restoration of missing tooth, implant placement is a widely used therapeutic modality. However, in some cases, there is low residual bone height or volume, caused by local trauma, tumor resection, or systemic conditions, necessitating bone regeneration prior to implant placement [4].

Over the last few decades, many different techniques and biomaterials, including guided tissue regeneration (GTR), guided bone regeneration (GBR), bone grafts of human, xenogenic or allogenic origins, growth factors, and various pharmacological agents, have been tested with the aim of regenerating periodontium and maxillary bone in vitro, in vivo, and in clinical settings but the results of the clinical trials have been, by and large, variable [5,6,7,8,9,10,11]. Current strategies for periodontal and bone regeneration are based on the fabrication of scaffolds which are biocompatible and can act as suitable vehicles for delivery of bioactive molecules (growth factors, drugs) or stem cells [12]. Not only does the scaffold material provide bulk mechanical support to the regenerating tissues but it also mimics the extracellular matrix (ECM) of tissues which directs the cell behavior to contribute towards the regenerative process [13]. In this context, control of inflammatory process has been suggested as sustained inflammation may impair regenerative therapeutic outcomes [14]. Membranes loaded with drugs such as ibuprofen (Ibu) and growth factors such as bone morphogenetic protein-2 (BMP-2) have already been tested in mice and have exhibited beneficial effects on wound healing and tissue regeneration [15,16]. Pre-clinical and clinical studies in the treatment of jaw bone defects are focused on bone substitution and regenerative approaches, the latter requiring novel experimental development and functionalization of bioactive molecules, different types of stem cells with synthetic biomembranes or scaffolds.

The association of transforming growth factor-beta 3 (TGF-β3) and dental pulp stem cells for peri-implant bone regeneration in an animal model of anterior implant repair showed promising results compared to mere bone substitution with bone powder [17]. Moreover, the trabecular bone was found to be having a superior bone density in the control group with surrounding osteoblasts arranged in clusters. Different types of stem cells (stem cells of human exfoliated deciduous teeth, human dental pulp stem cells, and bone marrow mesenchymal stem cells) have been compared for their ability to stimulate bone response in a model of calvarial defect in immunodeficient mice [18]. These stem cells were transplanted with a polylactic-polyglycolic acid (PLGA) scaffold and exerted similar bone regeneration abilities after 12 weeks of transplantation [18].

In humans, a recent study demonstrated positive results of a collagen-enriched xenogenic bovine bone mineral on post-operative volumetric bone alterations [19]. Another clinical technique for alveolar ridge preservation has been based on the association of xenogenic bone substitute with 10% collagen and covered with native bilayer collagen membrane [20]. Although a significant reduction of radiographic bone loss was observed with this technique. Nevertheless, these methods seem limited by the absence of biological bone and vascularization. To overcome this limitation, the use of mesenchymal stem cells (MSC) was clinically evaluated to treat maxillary bone defects following biopsies or osteolytic odontogenic benign tumors. Results showed promising outcome in terms of bone volume or density with MSC from autologous bone marrow on bone regeneration after biopsies or osteolytic lesions [21]. As the kinetics of in situ stem cells’ release cannot be controlled, functionalization of a synthetic PCL biomembrane with mesenchymal stem cells, as we proposed for the treatment of a maxillary bone lesion, may overcome such limitation.

Replacement of missing tooth by tooth tissue engineering has recently attracted much attention [22]. Therefore, besides periodontal tissue engineering, regenerating the entire missing tooth has also been attempted by tooth bioengineering. Vascularization and innervation are essential factors for homeostasis and response to noxious stimuli, determining the success of the bioengineered tooth [23]. Previous studies have shown that reassociations between dissociated mesenchymal cells and an intact epithelium from embryonic mouse molars (14th embryonic day, ED14) rendered it possible to obtain dental germs [24]. Their subcutaneous implantation in the mouse resulted in the formation and morphogenesis of molars that were vascularized but not innervated [25]. In 2014, Eap et al. synthesized ε-polycaprolactone (PCL) membranes by electrospinning and functionalized them with nerve growth factor (NGF) nanoreservoirs. By adding a trigeminal ganglion (TG) to the functionalized membrane and the germ, peripheral axons were detected in the pulp cavity as early as two weeks after implantation [26]. In another study, a TG was implanted with the germ to constitute a supply of nerve fibers, in conjunction with a systemic treatment with cyclosporine A (CsA) in the drinking water of mice [27]. This treatment allowed the subcutaneous development of vascularized and innervated molars as early as two weeks after implantation. CsA has immunomodulatory properties and stimulates nerve growth [28]. The side effects of the systemic administration of CsA, including renal dysfunction and cancers, have been widely reported and, thus, not negligible. To overcome this issue, local delivery of the molecule is more desirable, therefore, development of scaffold, such as biomembrane functionalized with nanoreservoirs of CsA, is of clinical interest with multiple therapeutic targets and has been successfully tested [29].

The objective of this review is to present new regenerative strategies based on controlled local delivery of active anti-inflammatory drugs and growth factors through functionalized membranes targeting each component of tooth and its supporting tissues.

## 2. Materials and Methods

### 2.1. Materials

Poly (d, l-lactic acid/glycolic acid) 50/50 polymer (PLGA; MW 24-38 KDa), under the commercial name Resomer^®^ RG 503, was purchased from Evonik Industries AG (Darmstadt, Germany). Polycaprolactone (PCL; MW 80 KDa) analytical grade, cyclosporine A (0.1 mg/mL), dexamethasone (used as HPLC internal standard), Pluronic^®^ F-68 surfactant, ethyl acetate (Class 3 solvent according to the pharmacopeia), acetonitrile, methanol (HPLC grade), and Ibuprofen (50 μg/mL) were all purchased from Sigma-Aldrich (St. Louis, MO, USA). BMP-2 (200 ng/mL) was acquired from Euromedex (Souffelweyersheim, France) and chitosan (Protasan UPCL 113, 500 μg/mL) from NovaMatrix (Sandvika, Norway).

### 2.2. Synthesis and Characterization of Cyclosporine A (CsA) Loaded PLGA Nanoparticles

Cyclosporine A loaded PLGA (PLGA/CsA) nanoparticles were prepared in a continuous microfluidic reactor using a PEEK-made interdigital micromixer (SIMM-V2, Slit Interdigital Micro Mixer, IMM, Mainz, Germany) by carrying out an oil-in-water (O/W) emulsification process followed by a solvent evaporation procedure as previously described [29]. Scanning electron microscopy (SEM, Inspect F50, FEI, Eindhoven, The Netherlands) was employed to determine the shape of the synthesized PLGA NPs.

### 2.3. PCL Scaffold Synthesis and Functionalization

PCL was dissolved in a mixture of dichloromethane/dimethylformamide (DCM/DMF 50/50 *v*/*v*) at 15% *w*/*v* and stirred overnight before use. A standard electrospinning set-up (EC-DIG apparatus, IME Technologies, Eindhoven, Netherlands) was used to fabricate the PCL scaffolds as described earlier [30]. The objective was to achieve nanoreservoirs distributed randomly on the surface of PCL nanofibers as shown in another study [30]. In our study, for some experiments, PCL scaffolds were incubated in a chitosan solution (chitosan, 500 μg/mL) for 15 min and rinsed with the buffer for 15 min. These scaffolds were then incubated in PLGA or PLGA/CsA NPs solution for another 15 min and, finally, thoroughly washed for 15 min, thus, constructing a ‘bilayer’ (chitosan/PLGA/CsA) on the fiber surface. Repetition of this protocol five times allowed the construction of (chitosan/PLGA/CsA)_5_, respectively. Even though this buffer solution provided high ionic strength to the media, the NPs remained strongly bound to the PCL electrospun nanofibers. For other experiments, (BMP-2/chitosan)_10_ and (Ibuprofen/chitosan)_3_ were built up on the PCL scaffold as described recently [15]. BMP-2 and ibuprofen remain protected and available for cellular activity due to their encapsulation in the nanoreservoirs of chitosan. Finally, Ibuprofen-functionalized PCL membranes (PCL/Ibu) were synthesized by mixing PCL pellets dissolved in DCM/DMF and Ibuprofen (10% of Ibu *w*/*w*) with TWEEN^®^ 80 and electrospinning process in a Yflow 2.2.D-500 electrospinner (Coaxial Electrospinning Machines/R&D Microencapsulation, Malaga, Spain) using the shell–core technique as described recently [16].

Scanning electron microscopy (SEM) was used to characterize fibers size and morphology of the different scaffolds as described earlier [15].

### 2.4. In Vivo Micro-Surgical Protocols

All experimental protocols fulfilled the authorization of the “Ministère de l’Enseignement Supérieur et de la Recherche” under the agreement numbers 01715.01 and 01715.02. The Ethics Committee of Strasbourg named “Comité Régional d’Ethique en Matière d’Expérimentation Animale de Strasbourg (CREMEAS)” specifically approved this study.

First, periodontitis was induced in mice by *Porphyromonas gingivalis*-infected ligatures to simulate disease condition comparable to human periodontitis as described previously [31]. To surgically treat the periodontal lesion, the test sites were treated with PCL/Ibu membrane [16].

Secondly, an intrabony periodontal lesion was created with a 0.5 mm round bur and a PCL/Ibu membrane was placed on the created bone lesion in such a manner that its ends could be blocked beneath the vestibular and palatal flaps. Bone level was evaluated by manual probing of the pocket depth and with the micro-computed tomography (micro-CT) analysis to confirm bone loss before initiating the treatment plan.

Thirdly, a maxillary bone lesion was created, under general anesthesia, in the diastemal area with a dental bur (0.8 mm) after gingival incision. On one side, bi-functionalized BMP-2/Ibuprofen or functionalized BMP-2 scaffold was implanted while the other side served as a control without scaffold or with non-functionalized scaffold for 30 and 90 days. The gingiva was closed with biological glue composed of enbucrilate (Histoacryl^®^, B. Braun, Rubi, Spain). To study the evolution of bone response, a longitudinal post-operative follow-up using micro-CT was conducted.

Finally, first mandibular molars were dissected from ICR mice (Charles River Laboratories, l’Arbresle, France) embryos at embryonic day 14 (ED14). Germs cultured on semi-solid medium reached the bell stage. For the innervation experiments, molars were cultured for six days on semi-solid medium as previously described [27], associated with a TG on PCL scaffolds (functionalized by chitosan/PLGA or chitosan/PLGA/CsA) for one night and implanted in the diastemal area. An incision was made up to the bone contact at the top of the alveolar crest in diastemal zone, in front of the first maxillary molar (M1). The bone lesion was obtained using a round bur (diameter 0.8 mm). Then, the cultured germ associated with TG on the CsA biomembrane was implanted and the lesion was closed with fibrin biological glue composed of enbucrilate (Histoacryl^®^, B. Braun, Rubi, Spain) for two and four weeks.

### 2.5. Histology and Indirect Immunofluorescence

For histology, samples were fixed for 24 h in 4% paraformaldehyde, decalcified in ethylenediaminetetraacetic acid (EDTA) at 37 °C for one week and embedded in paraffin. Serial sections (10 μm) were stained with hematoxylin/eosin or Gomori trichrome stain and observed on a Leica DM4000B microscope.

For the immunofluorescence, some samples were embedded in Tissue-Tek, frozen at −20 °C and sectioned (10 μm) using a cryostat (Leica, CM3000). Serial sections were rinsed with phosphate buffered saline (PBS), fixed for 10 min with 4% paraformaldehyde at 4 °C and treated as previously described [27], using anti-peripherin, anti-CD31 and anti-osteocalcin antibodies. Sections were observed with a fluorescence microscope (Leica DM4000B). 

## 3. Results

### 3.1. Characterization of the Biomembrane

**Figure 1:**
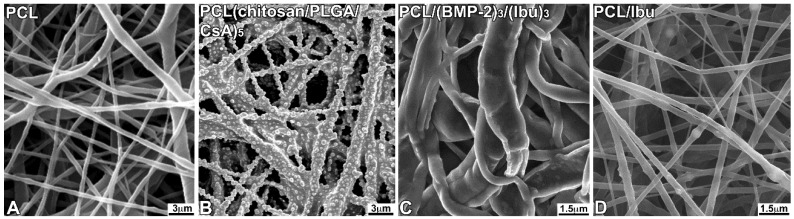
Scanning electron microscopy (SEM) observations of non-functionalized PCL scaffolds consisting of non-woven electrospun nanofibers (**A**), PCL scaffolds grafted with CsA-loaded PLGA nanoparticles (chitosan/PLGA/CsA)_5_ (**B**), with BMP-2/Ibuprofen (PCL/(BMP-2)_3_/(Ibu)_3_) nanoreservoirs (**C**) or with Ibuprofen (**D**). For the morphological study by SEM, the different scaffolds were fixed with 4% paraformaldehyde, dehydrated in successive baths of ethanol (25, 50, 75, 90, 100%) and treated with hexamethyldisilazane (HDMS). They were mounted on a supporting sample holder using carbon conductive adhesive, then, silver-coated and observed with a Philips XL-30 ESEM scanning electron microscope in conventional mode (high vacuum) with a Everhart-Thornley secondary electron detector.

The control nanofibrous structure (Figure 1A), the distribution of CsA (Figure 1B), BMP-2/Ibu nanoreservoirs (Figure 1C) and the PCL–Ibuprofen structure (Figure 1D) were characterized by SEM. The PCL scaffolds exhibited a nonwoven mesh like structure with a large surface area per volume ratio (Figure 1 A). The distribution of nanoreservoirs was random (Figure 1B). The morphology and fiber diameter distribution of the ibuprofen electrospun fibrous membrane showed that there were no beads in the fibrous structure and the fibers were uniform in size and interconnected in order to mimic the natural extracellular matrix (Figure 1D). The diameter of fibers was 374 ± 89 nm for the PCL/Ibu electrospun fibrous membrane.

### 3.2. Assessment of PCL Membrane Functionalized with Ibuprofen on Periodontal Wound Healing in Periodontitis-Induced Mouse Model

**Figure 2:**
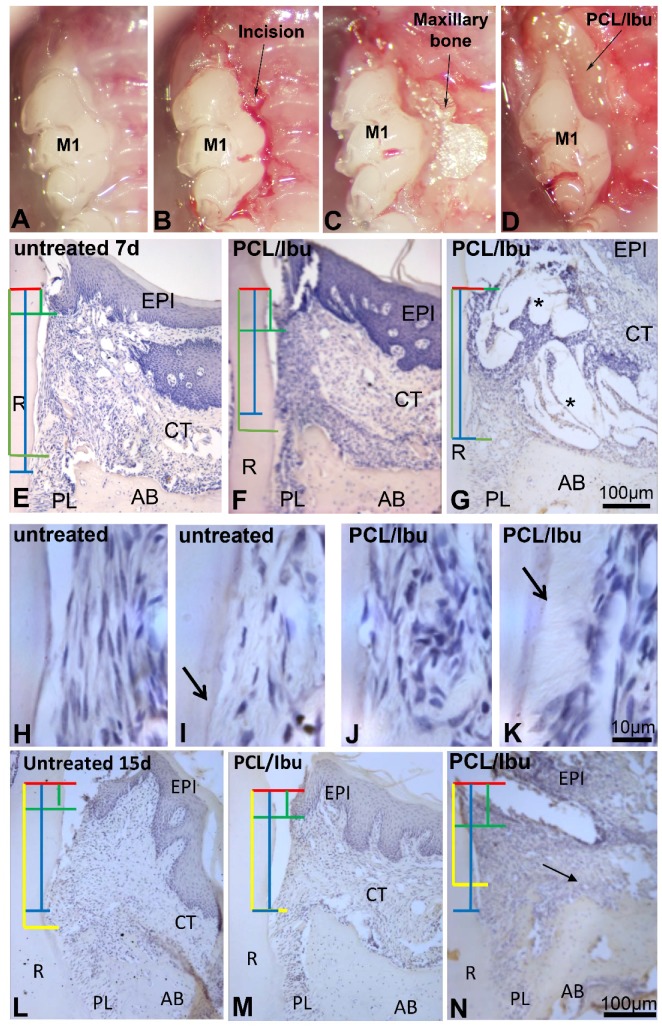
Periodontitis induced with *Porphyromonas gingivalis*-infected ligatures and treatment with PCL/Ibu membrane (**A**–**D**). (**B**) sulcular incision along the first and second maxillary molars, (**C**) raising the flaps for exposure and access, (**D**) surgical placement of PCL/Ibu membrane on the periodontal lesion, (**E**–**N**) histological view at 7 and 15 days. (**E**–**K**) histology of periodontal wound healing at 7 days and (**L**–**N**) at 15 days. Red line = cementoenamel junction, blue line = fibrous connective tissue attachment, green line = epithelial attachment, yellow line = bone level. After anesthesia, a slight incision to the bone crest contact was made to facilitate the first ligature placement at the junction between the gum and the tooth along the first and second molars (M1-M2) as previously described [16]. The thread was then blocked with a drop of glass ionomer (Fuji IIGC, GC, France, Bonneuil sur Marne, France). Sterilized black braided 6.0 silk threads (Ethicon, Auneau, France) were incubated in culture medium containing *P.gingivalis* in an anaerobic chamber for one day. *P.gingivalis*-soaked ligatures were placed around maxillary first and second molars. The ligatures were inspected and replaced (with freshly infected ones) thrice a week for a period of 40 days. An incision was performed along the sulcular margins of the first and second molars and extended anteriorly on the mesial aspect of the first molar to efficiently raise the flap to gain access. Ibuprofen-functionalized PCL membrane was punched with a 3 mm diameter cutter. The circular pieces of membrane were further divided into half to achieve a size appropriate enough to cover the lesion. The cut membrane was then placed into the periodontal pocket after raising the flap such that the membrane stays flat beneath the flap covering the lesion fully and the necks of the crowns (molars) partially, entering the inter-dental area as well. The flap was nicely repositioned to perform a suture on the flap while maintaining the membrane underneath [16]. AB: alveolar bone, CT: connective tissue, EPI: epithelium, PL: periodontal ligament, R: root. Stars showing PCL/Ibu membrane.

In periodontal wound healing at seven days (Figure 2E,H,I), inflammatory infiltrate could be observed in the control. Moreover, fibrous attachment primarily remained dominant but new cementum formation was also initiated (Figure 2I). More cementum formation was visualized in the test using PCL/Ibu (Figure 2K) compared to that in the control. The test also exhibited a better organization of the gingival tissue (Figure 2F,G). In fact, separate zones comprising dense cellular zone and collagen zone could be distinctively observed in the test. Membrane interposed between the cells and surrounded by inflammatory cells was shown in the test (Figure 2G). In the control, an increase in the cementum and bone formation was seen at 15 days of wound healing (Figure 2L–M) compared to that at seven days (Figure 2E–G). Epithelial attachment level was found to be improving while the fibrous attachment was observed to be replaced by epithelium and newly formed cementum. The differences between the control and test were less pronounced when the membrane persisted. Inflammation on the cervical margins of the persistent membrane could still be seen. Dense collagen bundles inserted on the bone away from the root surface were observed.

### 3.3. Assessment of PCL Membrane Functionalized with Ibuprofen on Periodontal Wound Healing in a Mesial Bone Defect Model

**Figure 3:**
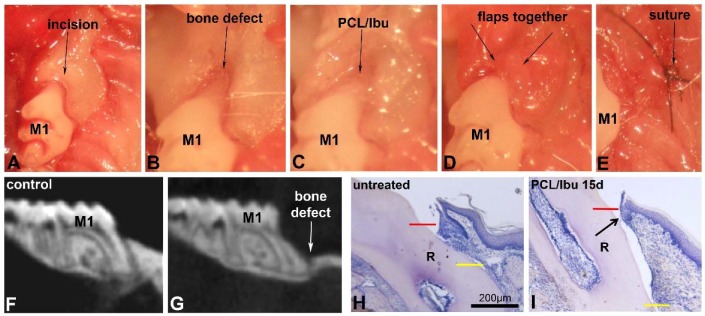
Surgical bone defect model and treatment with PCL/Ibu membrane (**A**–**I**). (**A**–**E**) demonstrate the surgical procedure for creating the mesial bone defect. After anesthesia, sulcular incision (**A**) was given along maxillary first molar and extended anteriorly on the mesial aspect of the first molar for efficient raising of palatal and vestibular flaps so that they do not hinder the bone drilling procedure. The exposed bone was drilled to create the intrabony defect (**B**). The bone over and around the mesial root of the first molar was removed. Constant irrigation with physiological saline was maintained to avoid overheating of the bur and the bone area concerned. The drilled bone was, later, nicely irrigated, cleaned, and dried to remove all the bone chips and debris. PCL/Ibu functionalized membrane was placed on the created bone lesion (**C**) in such a manner that its ends could be blocked beneath the vestibular and palatal flaps. Palatal and vestibular flaps were approximated covering the PCL/Ibu membrane underneath and sutured (9-0 ETHILON* Polyamide 6/6) or glued to retain the membrane underneath (**D**,**E**). (**F**) micro-CT view before the bony defect and (**G**) after bony defect. (**H**,**I**) Histology of periodontal wound healing at 15 days. Red line = cementoenamel junction, yellow line = bone level. (**I**) Arrow showing short epithelial attachment in test. M1: first upper molar, R: root.

A good bulk of the bone over and around the mesial root of the first molar was removed (Figure 3A–E) as confirmed by the micro-CT’s sagittal view (Figure 3F,G). Sagittal views of the histological sections compare the bone level and epithelial attachment level in the control (Figure 3H) and test (Figure 3I). Long junctional epithelium was found to be formed in the test (Figure 3I, arrow).

### 3.4. Maxillary Bone Regeneration Based on Nanoreservoirs Functionalized PCL with BMP-2 and BMP-2/Ibu

Surgery to study bone regeneration is the same as that depicted later in the text (Section 3.5). The only difference is that on one side, bi-functionalized BMP-2/Ibuprofen or functionalized BMP-2 scaffolds were implanted while the contra-lateral bone lesion served as a control without scaffold or with non-functionalized PCL scaffold for 30 and 90 days.

**Figure 4:**
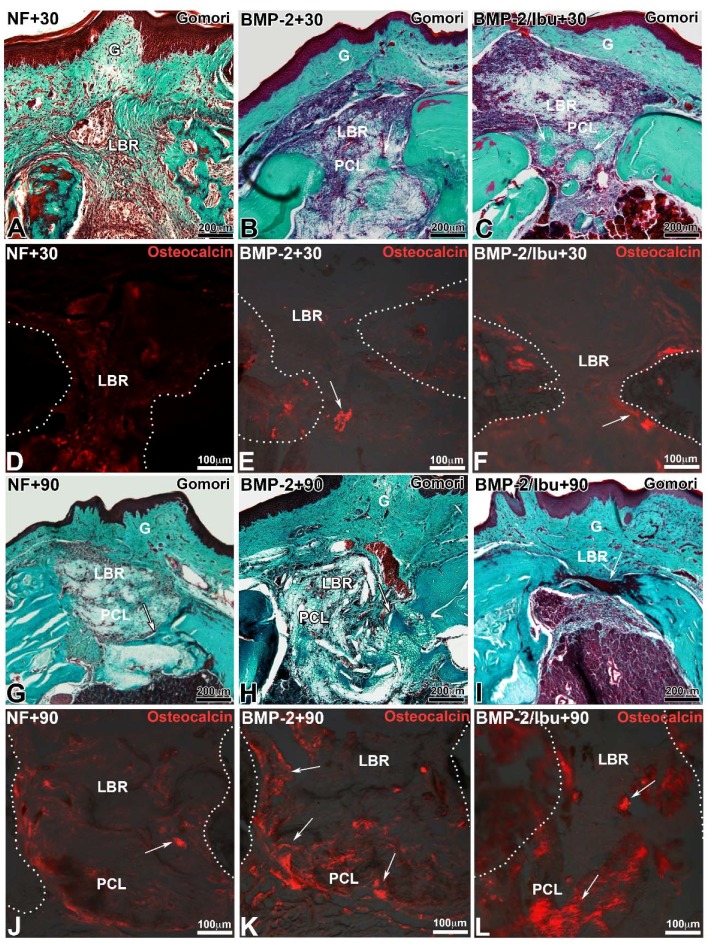
Trichrome of Gomori staining (**A**–**C**,**G**–**I**) and immunofluorescence for osteocalcin (**D**–**F**,**J**–**L**) after 30 (**A**–**F**) and 90 days (**G**–**L**) implantation of PCL (**A**,**D**,**G**,**J**), PCL/(BMP-2)_10_ (**B**,**E**,**H**,**K**) and PCL/(BMP-2)_10_/(Ibu)_3_ (**C**,**F**,**I**,**L**). Arrows indicated neoformed bone positive for osteocalcin. White dots indicate the limit of the maxillary bone. For the immunofluorescence, samples were embedded in Tissue-Tek, frozen at −20 °C and sectioned (10 μm) using a cryostat (Leica, CM3000). Serial sections were rinsed with PBS, fixed for 10 min with 4% paraformaldehyde at 4 °C and treated as previously described [27] using the rabbit anti-osteocalcin antibodies (Santa Cruz Biotechnology, dilution 1/200). Sections were observed with a fluorescence microscope (Leica DM4000B). G: gingiva, LBR: lesion with bone regeneration, PCL: scaffold.

Trichrome of Gomori stain and immunofluorescence for osteocalcin showed the degree of bone neoformation and closure of the bone lesion (Figure 4). At 30 days, the membrane is largely colonized by the cells (Figure 4A–C). For the mice treated with a non-functionalized membrane, little neoformed bone was observed at day 30 compared to the lesions treated with the membranes functionalized with BMP-2 or BMP-2/ibuprofen (Figure 4A). At 90 days, the bone bridge was thicker (Figure 4G–I). Neoformed bone showed trabeculations in different directions from the original bone. In lesions treated with PCL membrane or with PCL/BMP-2 (Figure 4G,H), areas of mineralization extended further and further tending to join the osseous banks at day 90 with BMP-2/ibuprofen (Figure 4I). A greater number of red blood cells was found around the functionalized PCL/BMP-2-treated lesion (Figure 4H) than the PCL/BMP-2/Ibu-treated lesion (Figure 4I). The control lesion appeared to be the least vascularized area. Osteocalcin antibody was used to demonstrate osteoblastic activity and bone neoformation (Figure 4D–F,J–L). After 30 days, immunofluorescence showed differences in bone formation according to the different scaffolds tested. Osteoblasts were visualized in the bone/scaffold interface (Figure 4E,F white arrows) for PCL/BMP-2 and PCL/BMP-2/Ibu scaffolds while with unfunctionalized PCL very few osteoblasts were detected (Figure 4D). After 90 days, there was a massive expression of this protein (Figure 4K,L, white arrows), allowing clear observation of distinctly differentiated osteoblasts in almost all microscopic fields within the scaffold area. These results corroborated the efficiency of biocompatible scaffolds in promoting new bone regeneration to repair maxillary bone defects.

**Figure 5:**
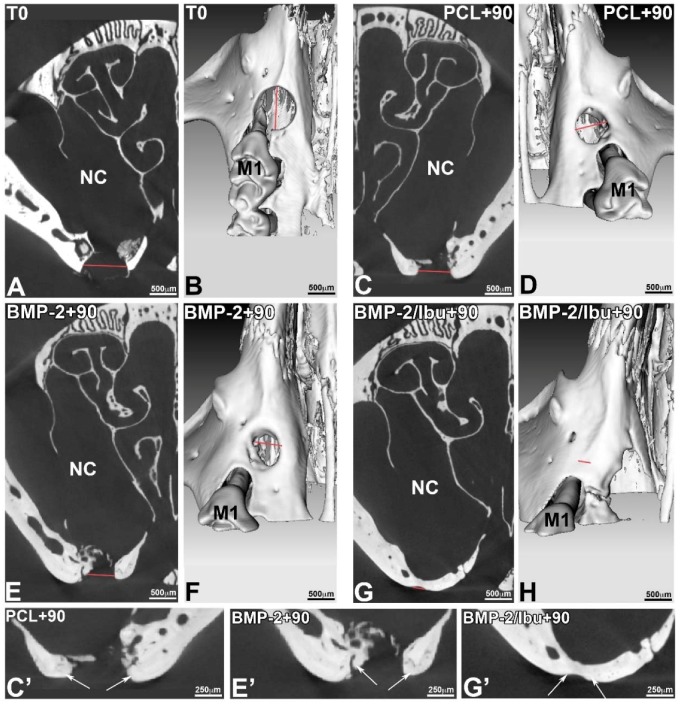
Micro-CT sections (**A**,**C**,**E**,**G**,**C’**,**E’**,**G’**) and 3D reconstructions (**B**,**D**,**F**,**H**) at T0 (**A**,**B**) and after 90 days of implantation of PCL (**C**,**D**,**C’**), PCL/BMP-2 (**E**,**F**,**E’**), and PCL/BMP-2/Ibu (**G**,**H**,**G’**). To study the evolution of bone response, we conducted an ex vivo longitudinal post-operative follow-up using micro-CT. The X-ray microtomography acquisitions were performed after 0 and 90 days. The size of the reconstructed isotropic voxel was 8 μm. M1: first upper molar, NC: nasal cavity.

The micro-CT (phoenix/X-ray, GE sensing & Inspection Technologies GmbH, Wunstorf, Germany) validated the position of the standardized lesion on the bone crest, in close proximity to the first molar. The sections acquired in micro-CT allowed visualizing the periosteal reaction at the base of the lesion, with regards to the nasal cavity (Figure 5C,E). This mechanism corresponds to a physiological osteoformation activity in response to the experimental surgical trauma. This micro-CT analysis confirmed that the bridge connecting the bone banks was mineralized (Figure 5G,G’). The micro-CT also measured the size of the initial bone defect (T0), which in this case corresponded to the diameter of the drilling bur used. On the 3D volume micro-CT reconstructions, the bony margins of the lesion were clear at T0 (Figure 5B), whereas at 90 days, the banks were more rounded, showing bone remodelling. We assessed the initial size of the lesion (Figure 5B) and observed the progressive bone response at 90 days with the BMP2-functionalized membranes and the non-functionalized PCL (Figure 5D,F) which does not lead to a closure of the bone banks. The 3D micro-CT reconstruction at 90 days in case of a bifunctionalized membrane with BMP-2 and ibuprofen (BMP-2/Ibu) showed a closure of the lesion, but the sections still showed that the bone formed was not as mineralized as the bone at the edges of the lesion (Figure 5C’,E’,G’). The thickness of the neoformed bone bridge formed was smaller compared to the initial situation (Figure 5G’).

### 3.5. Molar Bioengineering and Innervation After Bone Implantation Using CsA Functionalized Membrane

**Figure 6:**
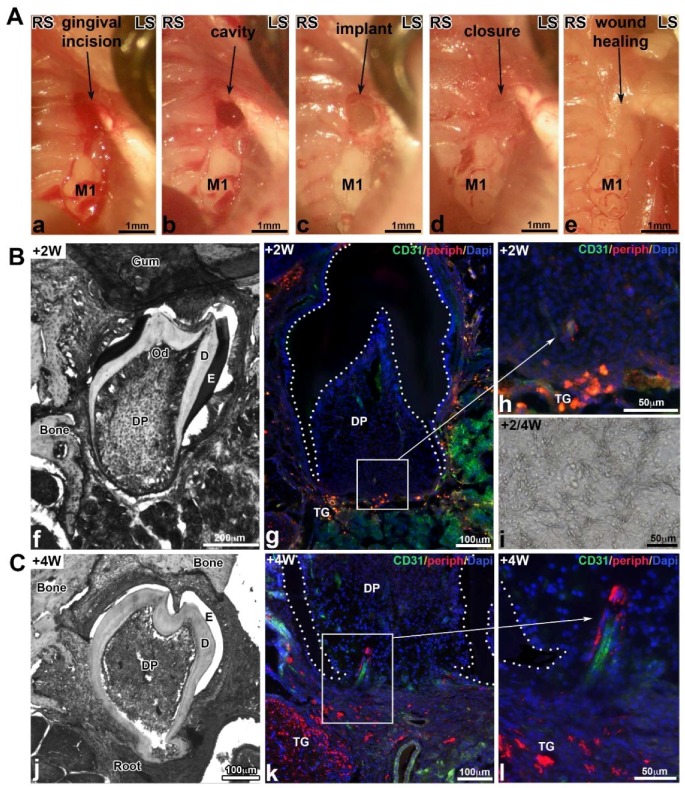
(**A**) Different stages of the microsurgery: incision of the gingiva (**Aa**), maxillary bone lesion obtained with a dental bur (500 μm) (**Ab**), implantation of the membrane with the bioengineered tooth and TG (**Ac**), closing of the gingiva with biological glue (**Ad**), and wound healing of the mucosa two weeks after implantation (**Ae**). (**B**,**C**) Histology, vascularization and innervation of bioengineered tooth implanted on PCL scaffolds functionalized with CsA-loaded PLGA nanoparticles (chitosan/PLGA/CsA)_5_ after two (**B**) or four (**C**) weeks of implantation. Samples were embedded in Tissue-Tek, frozen at −20 °C and sectioned (10μm) using a cryostat (Leica, CM3000). Serial sections were rinsed with PBS, fixed for 10 min with 4% paraformaldehyde at 4 °C. Some were stained with hematoxylin/eosin ((**Cf**),(**Ci**),(**Cj**)) or for the immunofluorescence as previously described using rabbit anti-peripherin (Abcam, dilution 1/600) and rat anti-CD31 (BD Pharmingen, dilution 1/100) antibodies [22] ((**Bg**),(**Bh**),(**Ck**),(**Ci**)). Cell nuclei were stained with 200 nM DAPI (Sigma-Aldrich Co, Darmstadt, Germany). D: dentin, DP: dental pulp, E: enamel, M1: first upper molar, Od: odontoblasts, TG: trigeminal ganglion.

An incision was made up to the bone contact at the top of the alveolar crest in diastemal area, in front of the M1 (Figure 6(Aa)). The bone lesion was obtained using a round bur (diameter 0.8 mm) (Figure 6(Ab)). Then, the cultured germ associated with a TG on the CsA biomembrane was implanted (Figure 6(Ac)) and the lesion was closed with a biological glue (Figure 6(Ad)), which allowed to cover the whole surgical site and promoted the wound healing. Two weeks after implantation, the mucosa was macroscopically and histologically closed (Figure 6(Ae)). Samples were recovered after two and four weeks of implantation (Figure 6B,C). Well-formed teeth were developed in the maxillary bone. The crown presented a normal morphogenesis with several cusps (Figure 6(Bf)) and the root formation was initiated after two weeks (Figure 6Bf) and further developed after four weeks (Figure 6(Cj)). Odontoblasts and ameloblasts were functional, they secreted predentin/dentin and enamel organic matrix, respectively (Figure 6(Bf),(Cj)). The PCL membrane was detected in contact with the bioengineered tooth (Figure 6(Bi)). Indirect immunofluorescence analysis two weeks after implantation revealed the presence of blood vessels positive for CD31 (Figure 6(Bg)) in the dental pulp and some nervous filaments positive for peripherin at the base of the tooth (Figure 6(Bh)). Four weeks following implantation, nerve fibers penetrated the dental pulp in the most apical region (Figure 6(Ck),(Cl)). In both cases, nerve fibers were associated with the blood vessels. After implantation with control PCL membranes ((chitosan/PLGA)_5_), bioengineered teeth were vascularized but not innervated (not shown) [22].

## 4. Discussion

Tissue regeneration is a pivotal field of research in dentistry, especially in regenerative endodontics or periodontology. The aim of the current therapeutic approaches is to regenerate lost tissues and several strategies have been developed and tested in this regard. Particularly, the use of bioactive scaffolds, such as membranes, has been widely studied [32].

In the context of periodontal and bone regeneration, synthetic membranes should combine both mechanical and biological properties to prevent their collapse within the defect and, ultimately, being capable of delivering ‘at-site’, the biomolecules or cells with controlled release to promote regeneration. To achieve this goal, electrospinning technique has been used to synthesize membranes from PCL [14,15,16,33]. PCL membranes are biocompatible, bioresorbable, and non-toxic [34,35]. Furthermore, they mimic efficiently the extracellular matrix supporting adhesion, differentiation, and cell proliferation. Interestingly, not only does their synthetic origin overcome the use of animal derived products but also exhibits desirable mechanical properties such as rigidity and low rate of resorption [36].

PCL membrane could also be utilized as an efficient drug delivery vehicle as described in this review with several interesting therapeutic applications for local delivery of certain bioactive agents such as anti-inflammatory or osteogenic molecules. Several strategies have been proposed to functionalize such scaffolds. Drugs, peptides, or other active molecules could be either inserted within the synthesized fibers through core-shell loading technique allowing a passive release of the compound during resorption of the fibers or by direct contact with the cells [16]. Nanoreservoir technology could also be used to deliver the active compounds to cells reaching tissues/organs in a controlled active manner as demonstrated for BMP2-PCL membrane [37]. In the context of periodontal diseases, evaluation of therapeutic efficacy should be assessed in both septic and aseptic conditions. Periodontitis is an inflammatory disease of infectious origin; therefore, it can be argued that concomitant to anti-inflammatory treatment, delivery of antimicrobial such as antibiotics would be of interest [38]. Here, we described two different models of periodontal destruction, one induced by infected ligature allowing to take into consideration the infectious nature of the disease, and the second one, where the lesion is mechanically induced by drilling in a depth-controlled manner. Thereafter, the test of new biomaterials or scaffolds for active compound delivery could be performed in a well-described environment.

The feasible synthesis of such PCL-membrane by electrospinning technique combining both core–shell and nanoreservoirs functionnalization will open new perspectives in the field of regenerative medicine. In this regard, combination of a passive anti-inflammatory drug release and nanoreservoir containing pro-regenerative molecules such as growth factors would be of great interest. The passive release of anti-inflammatory molecules may reduce the risk of persistent inflammation with concomitant active release of pro-regenerative drugs, promoting specific regeneration of the tissues. For instance, if combined in a such scaffold as described earlier, passive release of ibuprofen will decrease the inflammation leading to increased BMP-2 secretion by macrophages [39] while active loading of BMP-2 or other growth factor will directly promote in a specific manner, the regeneration of targeted tissue such as alveolar bone. This strategy could be developed with other growth factors combining osteogenic, osteoinductive, and angiogenic molecules such as vascular endothelial growth factor (VEGF) [40] or other signaling molecules such as hepatocyte growth factor (HGF), as an upregulation of VEGF and BMP-2 receptor via nuclear factor kappa B (NF-κB) has been shown for HGF, in cultured osteocytes and in vivo, promoting osteogenesis and neo-vascularization of tissue-engineered bone [41].

As described previously, the use of such scaffolds leads to the regeneration of small defects such as periodontal lesion as well as more significant bone destruction such as observed in the bone regeneration of critical size defect. However, the combination with stem cells, such as bone-marrow derived stromal cells, may be of interest to improve clinical outcomes [42]. Such strategies have already been evaluated and are already used clinically in orthopedic surgery, with functionalization based on multipotent mesenchymal stem cells [43]. However, it is of importance to describe the significant impact of the surgical technique used for membrane placement on regeneration related outcomes. Exposure of membrane has a potential detrimental influence on the outcome as observed for bone regeneration [44]. The full coverage of membrane by the flap is, therefore, mandatory. To our knowledge, no data are available regarding the effect of functionalized-PCL membrane exposure on the outcome of the therapy. Such parameters should be evaluated in the future to determine, more precisely, the potential of use of this type of biomaterial.

Tooth engineering has been the ultimate goal of regenerative dentistry for decades and many successful protocols have been described [45]. PCL membrane has been successfully tested to improve vascularization and innervation of the germ. For example, stem cells have the ability to stimulate axonal growth and are characterized by immunomodulatory properties. The concentration of nanoreservoirs can be adapted and the CsA release kinetics have already been the subject of a previous study [29]. In other medical domains, CsA has been used in the form of microspheres in hydrogel, which could be explored for tooth bioengineering [46].

In a previously reported work, we tried to implant reassociations in alveolar bone on M1 or M2 extraction sites but the tooth germ did not develop and was resorbed. We assume that this failure was due to a difference in bone type and bone healing metabolism. The hypothesis established that natural bone healing occurred more rapidly at the extraction site (pulp bleeding, alveolar bone, presence of mesodermal cells, and odontogenic mesenchymal stem cells) than at the level of a diastemal bone lesion in the diastemal zone (basal bone with poor vascularization). Hence, it was necessary to combine tooth regeneration techniques with bone regeneration strategies to prepare the implant site in the best manner possible. Besides its effect on osteogenesis, the membrane allowed isolation of the lesion from the nasal cavity, which was otherwise mostly approached during the milling process, in this murine model. This exposure of the lesion to bacteria of the nasal cavity could slow bone healing and this risk is greater in the absence of the membrane. Interestingly, in such model, fibrin glue could be used to protect the surgical site and maintain the membrane on site since it does not interfere with the underlying bone healing [47].

## 5. Conclusions

The development of regenerative nanomedicine illustrated by the synthesis and characterization of bioactive scaffolds such as membranes will open new therapeutic conservative approaches aiming to maintain, at long-term, the existing teeth and also, when required, to restore esthetics and function of missing teeth without exogenous devices such as dental implants.
